# The Inflammatory Bowel Disease Transcriptome and Metatranscriptome Meta-Analysis (IBD TaMMA) framework

**DOI:** 10.1038/s43588-021-00114-y

**Published:** 2021-08-20

**Authors:** Luca Massimino, Luigi Antonio Lamparelli, Yashar Houshyar, Silvia D’Alessio, Laurent Peyrin-Biroulet, Stefania Vetrano, Silvio Danese, Federica Ungaro

**Affiliations:** 1 Department of Biomedical Sciences, Humanitas University, Pieve Emanuele, Milan Italy; 2 IBD Center, IRCCS Humanitas Research Hospital, Rozzano, Milan Italy; 3 PhoenixLAB, Lodi, Italy; 4 Inserm NGERE, University of Lorraine, Vandoeuvre-les-Nancy, France; 5 Nancy University Hospital, Vandoeuvre-les-Nancy, France

**Keywords:** Inflammatory bowel disease, Computational platforms and environments, RNA sequencing

## Abstract

Inflammatory bowel disease (IBD) is a class of chronic disorders whose etiogenesis is still unknown. Despite the high number of IBD-related omics studies, the RNA-sequencing data produced results that are hard to compare because of the experimental variability and different data analysis approaches. We here introduce the IBD Transcriptome and Metatranscriptome Meta-Analysis (TaMMA) framework, a comprehensive survey of publicly available IBD RNA-sequencing datasets. IBD TaMMA is an open-source platform where scientists can explore simultaneously the freely available IBD-associated transcriptomics and microbial profiles thanks to its interactive interface, resulting in a useful tool to the IBD community.

## Abstract (web summary)

Massimino et al. propose the Inflammatory Bowel Disease Transcriptome and Metatranscriptome Meta-Analysis (IBD TaMMA) framework, an open-source platform for expediting the investigation of IBD-specific transcriptomics and metatranscriptomics signatures.

## Main

Inflammatory bowel disease (IBD), including ulcerative colitis (UC) and Crohn’s disease (CD), is a class of multifaceted chronic inflammatory gut disorders characterized by an uncontrolled, resolution-failing inflammation that leads to bowel damage^1^. In recent decades, numerous omics studies have focused on understanding IBD pathogenesis. Despite the high amount of RNA-sequencing (RNA-seq) data produced to help preclinical and clinical research, such studies are hard to integrate due to their experimental variability and different analytic approaches^2^. To exploit the efforts made over the years by IBD experts in the field of next-generation sequencing (NGS), we here introduce a meta-analysis web app, the IBD Transcriptome and Metatranscriptome Meta-Analysis (TaMMA) platform. IBD TaMMA is a comprehensive survey of publicly available RNA-seq datasets from IBD-derived and control samples across different tissues, all analyzed with the same pipeline and batch-corrected for data harmonization and simultaneous comparison among the different studies. This tool, featuring increased statistical power due to its augmented sample size, provides to the scientific community a user-friendly, open-source platform where data-mining of the IBD-associated transcriptome and metatranscriptome can be faster and statistically more powerful than each single study alone, resulting in a useful tool for the IBD community.

## Results

Various meta-analyses of gene expression profiles of patients with IBD from microarray datasets have already identified dysregulation of the expression of genes encoding for several inflammatory factors and RNA-binding proteins^3–5^. However, these studies focused on a limited number of genes and lacked not only whole-transcriptome but also metatranscriptome profiling. The latter has recently emerged as a successful approach to uncover novel gut-populating microbial entities^6^.

**Fig. 1:**
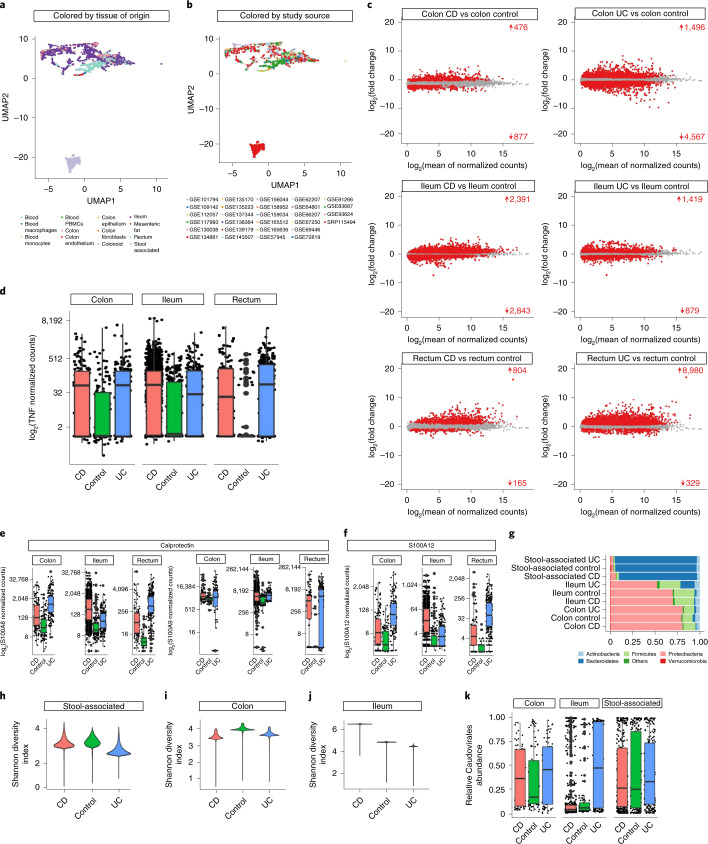
IBD TaMMA overview. **a**,**b**, Multidimensional scaling of the human whole transcriptome by UMAP from patients with UC and CD, as well as healthy (control) participants. **c**, MA plots showing the differential gene expression results, expressed as log_2_(fold change) between the indicated comparisons (M) as a function of log_2_(average gene expression) (A). Red dots represent genes being differentially expressed with high statistical significance (false discovery rate (FDR) < 1 × 10^−10^). The number of differentially expressed genes and their trends are indicated in red. **d**, Box plots showing differential tumor necrosis factor (TNF) normalized expression among UC and CD, as well as healthy ileum, colon and rectum. **e**,**f**, Box plots showing differential calprotectin (S100A8 and S100A9) (**e**) and S100A12 (**f**) encoding gene normalized expression among UC and CD, as well as healthy (control) ileum, colon and rectum. **g**, Bar plots showing the relative abundance of the indicated bacterial phyla in stools, ileum and colon from patients with UC and CD, and healthy participants (control). **h–j**, Violin plots showing Shannon diversity indices among CD, healthy (control) and UC in stools (**h**), colon (**i**) and ileum (**j**). **k**, Box plots showing relative Caudovirales order abundance in colon, ileum and stools from healthy participants (control) and patients with CD, and UC. All box plots represent the sample distribution with median, minimum, maximum, first and third quartiles. An interquartile range of 1.5 is used to define outliers. Statistical differences between groups were calculated by analysis of variance with Tukey’s honestly significant difference post hoc test for multiple comparisons. Differences with adjusted *P* ≤ 0.05 were considered significant. For complete statistics, see Supplementary Table 2. Source data

To provide a wider picture of the whole transcriptome and metatranscriptome at different tissue and cell levels in patients with both UC and CD, we collected and analyzed publicly available RNA-seq datasets. As this involved 26 independent studies, we predicted an experiment-dependent bias, which we counteracted with ComBat^7^, a batch-correction algorithm that is well established in transcriptomics for adjusting unwanted sources of variation in the context of high-throughput experiments^8^, following the source and tissue of origin. We also tried to batch-correct the different library construction strategies, but their variance was already fully explained by the source study. The meta-analysis performed was used as the core to design the IBD TaMMA web app (Supplementary Fig. 1a), intended as a set of analyses displayed in a web browser, which allows quick access to differential gene expression and Gene Ontology functional enrichment results for the different conditions. Sample dispersion within the Uniform Manifold Approximation and Projection (UMAP), easily accessible through the IBD TaMMA platform, shows clustering following the tissue of origin but not the source study (Fig. 1a,b), indicating successful data harmonization. Consistently, housekeeping gene expression levels were found to be comparable across the different tissues and conditions (Supplementary Fig. 1b).

Notably, owing to the lack of patient metadata in the majority of studies integrated into IBD TaMMA, we could perform neither stratification nor clustering based on patient characteristics (age and gender). Similar limitations are found in previously published studies, although they offered important contributions for the development of innovative IBD-counteracting therapies. To mitigate limitations related to the absence of patient metadata, we first assessed whether this platform confirmed previously established IBD-specific features. Our platform indeed pinpoints strong differential gene expression among UC, CD and healthy (control) groups in the ileum, colon and rectum, as shown in Fig. 1c. Of note, IBD-specific proinflammatory signatures were confirmed. Specifically, by comparison with healthy tissues, IBD-derived intestinal tissues displayed increased expression levels of tumor necrosis factor-α (*TNF*), interferon-γ (*IFNG*), interleukin-12B (*IL12B*), integrin-α4 (*ITGA4*) and integrin-β7 (*ITGB7*), encoding for proteins known to be drivers of chronic inflammation and thus exploited as therapeutic targets for patients with IBD^9^ (Fig. 1d, Supplementary Fig. 1c and the ‘Old evidence from the literature’ tab at IBD TaMMA). Similarly, S100 calcium-binding protein A8 and A9 (*S100A8* and *A9*) transcripts encoding for the two subunits of the fecal biomarker calprotectin^10^ and the recently emerged *S100A12*^10^ were increased in intestinal samples from CD and UC as compared to the healthy (Fig. 1e,f) groups. These results are well in line with most of the studies reporting these molecules as biomarkers of inflammation in patients with IBD^10^. Additionally, epithelium- and pro-angiogenic-related biological processes, known to be altered during gut chronic inflammatory disorders^11–14^, were also found to be dysregulated in both UC and CD colon versus control colon, as well as in CD ileum versus control ileum (Supplementary Fig. 1d and ‘Old evidence from the literature’ tab at IBD TaMMA).

Metatranscriptomics performed on IBD and healthy stools^15^ paralleled previous metagenomics analysis, confirming the Bacteroidetes and Firmicutes phyla, followed by Actinobacteria and Proteobacteria, as the main colonizers of the fecal microbiota^16^ (Fig. 1g, upper bars). IBD TaMMA also highlighted IBD and healthy intestinal samples to be colonized by the same phyla, although with different proportions (Fig. 1g, lower bars). Moreover, decreased intestinal microbiota diversity, a well-known feature of IBD pathogenesis^1^, was confirmed in IBD stools as compared to healthy stools (Fig. 1h), paralleled by decreased diversity also in the colon and ileum from the UC group and in the colon from the CD group (Fig. 1i,j). Interestingly, CD ileum showed increased microbiota diversity compared to the other groups (Fig. 1j), providing an insight into the disease location-dependent microbiota composition in patients with CD. Of note, IBD TaMMA also confirmed virome dysbiosis, with the expansion of Caudovirales in both pediatric IBD and UC samples^17,18^ (Fig. 1k), as well as increased levels of the Herpesviridae family in IBD-derived samples and of the Hepadnaviridae family in UC ileum as compared to the healthy, as previously reported^19,20^ (Supplementary Fig. 1e,f). Among the Herpesviridae-belonging viruses, cytomegalovirus (CMV) genus infection was previously associated with complicating UC, and its presence correlated with increased colectomy and mortality rates in patients with UC^21^. CMV includes different species of human beta herpesvirus species whose transcripts from IBD TaMMA were found to be upregulated in patients with IBD. Specifically, human beta herpesvirus 5 was highly abundant in UC and CD colon and CD ileum as compared to healthy tissues (Supplementary Fig. 1g and ‘Old evidence from literature’ tab at IBD TaMMA). These data support previously published evidence and indicate that high levels of the CMV genus-belonging beta herpesvirus 5 are associated with intestinal inflammation.

We then attempted to uncover novel aspects of the IBD-derived samples. We present evidence generated by meta-analysis of the archaeome and mycome composition. Archaeome analysis by TaMMA revealed that the archaea Nitrosophaerales, Haloferacales, Natrialbales and Thermococcales were among the most abundant archaea orders in the CD ileum, whereas the most abundant orders in UC ileum were Methanococcales, Methanobacteriales, Methanosarcinales and Methanomicrobiales, evidencing the differences between the two diseases in the ileal part. Interestingly, members of Methanomicrobiales were also found higher in UC colons, where it was the sole archaeal order to be statistically significant, while no differences were observed in colons from patients with CD. From these insights, we can conclude that each intestinal tract may display differential abundances of archaea, not only reflecting the specific gut tract, but also specific disease conditions (‘New evidence by TaMMA’ tab).

Regarding the mycome profile, CD and UC ileum both feature an increased abundance of Glomerellales, Tremellales and Hypocreales while also featuring a decreased abundance of Schizosaccharomycetales. Some orders were differentially abundant in the two conditions. Saccharomycetales, Ustilaginales, Malasseziales, Eurotiales, Mycosphaerellales and Magnaporthales were found to be differentially abundant exclusively in UC ileum, while Saccharomycetales, Ustilaginales and Sordariales were dysregulated only in CD ileum. Interestingly, different from the ileum, very few orders were found to be differentially abundant in the colon, perhaps resembling the different immune competence of the two tissues^20^. In conclusion, the mycobiome composition is gut tract- and disease-specific, opening additional horizons for IBD-associated microbiota diversity (‘New evidence by TaMMA’ tab).

It is noteworthy that, during the analysis, most of the human unmapped reads failed to be classified by metatranscriptomics profiling and therefore were considered as NGS dark matter. These data have been submitted to a data repository (given in the ‘Data availability’ statement) as we believe these data can also contribute to the understanding of gene and microbial entities not yet known but that may be the aim of future investigations (to discover new microbial entities).

In conclusion, together, these pieces of evidence propose IBD TaMMA as a platform that can confirm well-known features of IBD pathogenesis, hopefully resulting in a useful open-source tool for uncovering further insights into personalized diagnosis and prognosis upon treatment.

## Discussion

Numerous whole-transcriptome analyses of IBD samples have been performed, but a platform where these data can be browsed and compared is currently lacking. The IBD TaMMA web app introduces an integrative analysis of all IBD-related publicly available RNA-seq datasets and has been designed to have a graphical interface that allows users to interact with it, helped by a guide, icons and dropdowns selecting specific analysis and comparisons. However, owing to the lack of patient metadata, many clinically relevant aspects cannot be confirmed, such as the correlation between CMV infection and increased risk of colectomy in patients with UC^21^. Of course, including other characteristics will reinforce the web app, and we propose to update it soon as they are available in other datasets. We thus hope that more clinical metadata will be added over time, finally providing more insights into the different aspects of chronic intestinal inflammation.

As IBD TaMMA is a visualization rather than an analysis web app, users cannot upload or analyze their data independently. However, although future versions of IBD TaMMA may be developed as analysis web apps, in the meantime we encourage users to ask for the analysis of new datasets through the dedicated link https://github.com/Humanitas-Danese-s-omics/ibd-meta-analysis-data/issues.

As future steps for the IBD TaMMA, additional advantages will include the analysis of samples from IBD patients with extraintestinal manifestations (https://www.crohnscolitisfoundation.org/what-is-ibd/extraintestinal-complications-ibd). This may help understand the mechanism through which some patients may experience IBD-related complications.

Updated versions of IBD TaMMA will include the analysis of differential gene abundances for the microbiota, adding valuable information, as well as the realization of a dedicated TaMMA-like platform comprehensively analyzing IBD along with other immune-mediated disease-derived samples, which may expedite the discovery of shared features.

IBD TaMMA will also be implemented with other omics analyses, such as genomics and proteomics, and, thanks to the multi-omics analysis approach^22^, we will show the real variance explained by each omics, instead of using a single dataset at a time (for example, archaea only), which would consider only a part of the total picture. This is indeed the direction in which we are proceeding for future releases of the updated versions of IBD TaMMA, offering the opportunity to propose new hypotheses and insights for a better comprehension of IBD pathogenesis and the development of personalized treatments.

## Methods

### RNA-sequencing data

The pipelines for RNA-seq data download and analysis were designed with Snakemake v6.4.1^23^. FASTQ reads from 3,853 RNA-seq data (6.5 terabytes, 13 tera base pairs) were searched in NCBI GEO/SRA using the following search query: ‘(crohn OR colitis OR ibd) AND expression AND sequencing AND sapiens’, last queried on 6 March 2021. All freely available IBD-related datasets were included. Only low-quality reads within samples were discarded before the analysis pipeline.

FASTQ file download from NCBI SRA and initial quality control (QC) filtering were performed with fastq-dump v2.11.0 (https://trace.ncbi.nlm.nih.gov/Traces/sra/sra.cgi?view=toolkit_doc&f=fastq-dump). Additional quality checks and adaptor trimming were performed with FastQC v0.11.9 (https://www.bioinformatics.babraham.ac.uk/projects/fastqc/) and Trimmomatic v0.39^24^.

Read mapping to the human reference genome (GRCh38 primary genome assembly), finalized with GENCODE v35 gene annotations^25^, and gene quantification were performed with STAR v2.7.9a^26^. Post-mapping QC was performed with RSeQC v4.0.0^27^ and MultiQC v1.10.1^28^.

### Meta-analysis

Because these data came from 26 different studies from different laboratories, we counteracted the presumptive bias by batch correction in accordance with source (batch covariate) and tissue of origin (explaining other possible covariance). Batch-effect detection and correction were performed with ComBat^7^, within the Surrogate Variable Analysis v1.8 R package (https://bioconductor.org/packages/release/bioc/html/sva.html). Correction was performed with the following parameters: sva::ComBat(dat=raw_counts, batch=source, mod=tissue), where raw_counts is the merged count matrix, and the source of origin was used as a batch covariate and the tissue type as a possible other covariate.

Once the gene counts were adjusted, samples were divided into groups in accordance with the tissue of origin and patient condition before differential expression analysis and Gene Ontology functional enrichment. Differential human gene expression and differential species/family/order abundance analyses were performed with DESeq2 v1.32.08^29^. Multi-core parallelization was achieved with BiocParallel v1.26.0 (https://github.com/Bioconductor/BiocParallel). Gene-level annotations were managed by ensembldb v2.16.09 and EnsDb.Hsapiens.v86 v2.99.0 (http://bioconductor.org/packages/release/data/annotation/html/EnsDb.Hsapiens.v86.html).

The statistics for human differential gene expression, Shannon diversity and viral entity relative abundance are shown in Supplementary Table 2. Comparisons with FDR < 1 × 10^−10^ are considered statistically significant.

Functional enrichment analysis of the Gene Ontology biological process was performed with GeneSCF v1.110^30^, using differentially expressed genes with FDR < 1 × 10^−10^.

Finally, the reads failing to map to the human genome were subjected to metatranscriptomics profiling by taxonomic classification of archaeal, bacterial, eukaryotic or viral genes. Taxonomic classification of human genome-unmapped reads by exact k-mer matching against archaeal, bacterial, eukaryotic or viral genomes was performed with Kraken2 v2.1.211^31^. Bacterial species Spearman diversity and Simpson dominance indices were calculated with vegan v2.5 (https://github.com/vegandevs/vegan).

Low-dimensional embedding of high-dimensional data was performed with umap v0.2.7.0 (https://cran.r-project.org/web/packages/umap/vignettes/umap.html).

Data carpentry was performed in R with tidyverse v1.3.1 (https://www.tidyverse.org/) and Python with NumPy v1.20.3 (https://numpy.org/) and pandas v1.2.4 (https://pandas.pydata.org/). Plots in the figures were created with ggplot2 v3.3.312.

### Clinical metadata

Even if of valuable importance, clinical data are often missing from the analyzed datasets. However, because we recognize that, even if partial, this information could be useful, we take into account disease stage (*n* = 21), Mayo score (*n* = 206), Crohn’s disease activity index (CDAI, *n* = 155) and pediatric ulcerative colitis activity index (PUCAI, *n* = 206) as information deserving annotation. This can be retrieved in the metadata table and the ‘Color by’ dropdown.

### Web app design

The web app was developed with Dash v1.20.0 (https://dash.plotly.com/) and Plotly v4.14.3 (https://plotly.com/), stored in GitHub and run in Heroku. Programmatic access to the data tables is performed by Requests v2.25.1 (https://docs.python-requests.org/). Programmatic access to the data tables is performed by Requests v2.25.1 (https://docs.python-requests.org/).

The web app will be updated as new datasets become available. Users can suggest missing studies by clicking on the ‘Suggestions’ link in the TaMMA footnotes.

The complete guide on how to use the TaMMA web app is available at https://ibd-tamma.readthedocs.io/.

### Statistics and reproducibility

Statistical differences between groups in Fig. 1d–k and Supplementary Fig. 1b–c,e–g were calculated by analysis of variance with Tukey’s honestly significant difference post hoc test for multiple comparisons. Differences with adjusted *P* ≤ 0.05 were considered significant.

### Ethics statement

Please refer to the original articles for the ethical approval of the human studies mentioned in this paper.

### Supplementary information

Supplementary Information Supplementary text and Fig. 1.

Supplementary Table 1 Table describing the input datasets and their references.

Supplementary Table 2 Statistics of differential gene expression analyses and relative abundance comparisons mentioned in the manuscript.

Supplementary Data 1 Normalized gene counts, endothelium-related gene ontologies categories and virus relative abundances.

### Source data

Source Data Fig. 1 Fully annotated UMAP, differential gene expression results, bacteria order relative abundance, Shannon diversity indices, normalized gene counts and Caudovirales relative abundance.

## Data Availability

FASTQ reads were mined from the NCBI GEO/SRA data repositories. Study IDs with their respective links and references are provided in Supplementary Table 1. The relevant datasets mentioned in this paper are available in the summary and metadata tabs within the IBD TaMMA web app, and the described results are available in the literature tab within the app. The underlying data for the web app are available at https://github.com/Humanitas-Danese-s-omics/ibd-meta-analysis-data and in the Open Science Framework repository^32^. Human unmapped FASTQ reads that failed to be classified by metatranscriptomics profiling have been considered as NGS dark matter. The IBD TaMMA NGS dark matter is available at https://dataverse.harvard.edu/dataverse/tamma-dark-matter. Source data are provided with this paper and in the Open Science Framework repository^32^.

## Code availability

The code is stored in GitHub at https://github.com/Humanitas-Danese-s-omics/ibd-meta-analysis and in the Open Science Framework repository^32^, and running at https://ibd-meta-analysis.herokuapp.com/.
