# Author Correction: The Inflammatory Bowel Disease Transcriptome and Metatranscriptome Meta-Analysis (IBD TaMMA) framework

**DOI:** 10.1038/s43588-021-00146-4

**Published:** 2021-09-29

**Authors:** Luca Massimino, Luigi Antonio Lamparelli, Yashar Houshyar, Silvia D’Alessio, Laurent Peyrin-Biroulet, Stefania Vetrano, Silvio Danese, Federica Ungaro

**Affiliations:** 1 Department of Biomedical Sciences, Humanitas University, Pieve Emanuele, Milan Italy; 2 IBD Center, IRCCS Humanitas Research Hospital, Rozzano, Milan Italy; 3 PhoenixLAB, Lodi, Italy; 4 Inserm NGERE, University of Lorraine, Vandoeuvre-les-Nancy, France; 5 Nancy University Hospital, Vandoeuvre-les-Nancy, France

**Keywords:** Inflammatory bowel disease, Computational platforms and environments, RNA sequencing

Correction to: *Nature Computational Science* 10.1038/s43588-021-00114-y, published online 20 August 2021.

In the version of this Brief Communication initially published, there were errors in author affiliations. Specifically, affiliation 2 (IBD Center, Humanitas Clinical and Research Center – IRCCS, Rozzano, Milan, Italy) has been corrected to read: “IBD Center, IRCCS Humanitas Research Hospital, Rozzano, Milan, Italy.” Further, Luca Massimino was missing a footnote to affiliation 2, while Luigi Antonio Lamparelli’s affiliations included affiliation 1 (Department of Biomedical Sciences, Humanitas University, Pieve Emanuele, Milan, Italy) in error. The footnotes have been restored and removed, respectively. The changes have been made to the online version of the article.

