# Supplementary material for: The Inflammatory Bowel Disease Transcriptome and Metatranscriptome Meta-Analysis (IBD TaMMA) framework
Source: Nat Comput Sci. 2021 Aug 20;1(8):511–5. doi: 10.1038/s43588-021-00114-y (PMC10766544; doi:10.1038/s43588-021-00114-y)
Supplement: Supplementary file 1 — Supplementary text and Fig. 1. [file 43588_2021_114_MOESM1_ESM.pdf]

nature portfolio

https://doi.org/10.1038/s43588-021-00114-y

Supplementary information

The Inflammatory Bowel Disease Transcriptome and Metatranscriptome Meta-Analysis (IBD TaMMA) framework

In the format provided by the authors and unedited

Supplementary text

To the best of our knowledge, no interactive web app for navigation of omics data related to IBD samples is currently available as an open source to the scientific community. Other similar platforms are available for cancer studies (GENT2; GDC). The main functions of both platforms are the visualization of the differential and tissue-wide expression of the gene(s) of interest, while no more composite analyses (similar to the multidimensional scaling by UMAP in IBD TaMMA) are easily accessible and no meta-transcriptomics is provided.

The resourceful aspect of TaMMA is its interactive nature as a platform, where users can easily "play" with the data, finally obtaining personalized graphs for data visualization to address their specific questions.

Being the IBD TaMMA progressively updated as soon as the new studies will become freely available, we encourage users to suggest their studies to be analyzed within TaMMA through a dedicated "Suggestions" link in the TaMMA footnotes (https://github.com/Humanitas-Danese-s-omics/ibd-meta-analysis-data/issues).

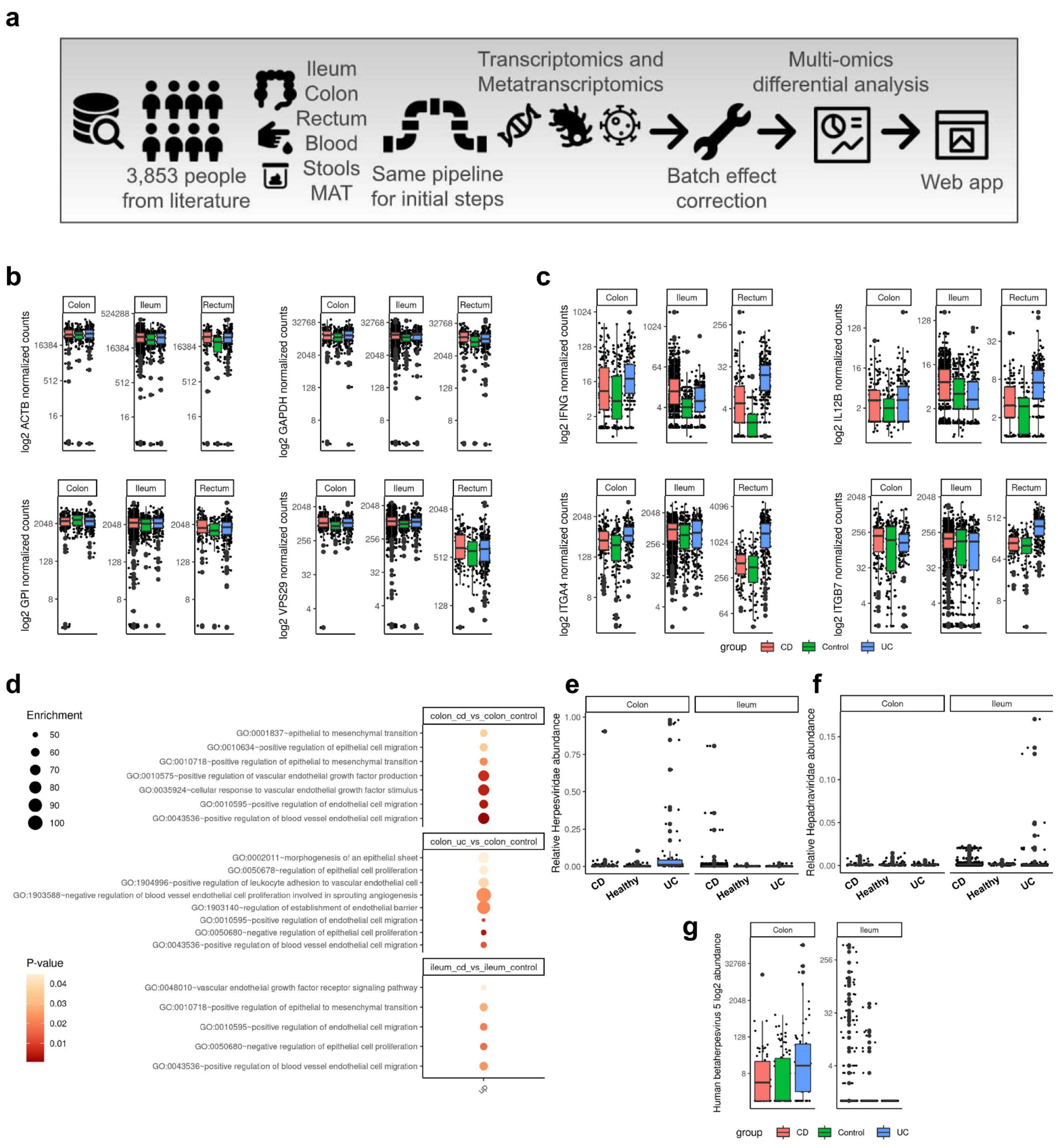

Supplementary Figure Legend

Supplementary Figure 1. IBD TaMMA overview. (**a**) Schematic representation of the entire IBD TaMMA workflow. This image has been designed using resources from https://streamlineicons.com. (**b**) Box plots showing comparable gene expression levels of the housekeeping Actin-beta (*ACTB*), Glyceraldehyde 3-phosphate dehydrogenase (*GAPDH*),

Glucose-6-Phosphate Isomerase (*GPI*), and Vacuolar protein sorting-associated protein 29 (*VPS29*) among ileum, colon, and rectum from CD, UC and healthy human subjects (Housekeeping from Eisenberg and Levanon, 2013). (**c**) Box plots showing normalized expression levels of Interferon-gamma (*IFNG*), Interleukin 12B (*IL12B*), Integrin alpha 4 (*ITGA4*), Integrin beta 7 (*ITGB7*), in the ileum, colon, and rectum from CD, UC, and healthy human subjects.. (**d**) GO plot showing modulation of angiogenic and epithelial function-related biological pathways in UC and CD colon by comparison with the control and in CD ileum compared to the healthy. (**e,f**) Box plots showing the results of metatranscriptomics performed on CD, UC, and healthy ileum, colon, and rectum highlighting the increased relative abundance of *Herpesviridae* family in CD ileum and UC colon by comparison with the healthy (e), as well as the relative abundance of *Hepadnaviridae* family in the ileum from UC, CD, and healthy patients (f). (**g**) Box plots showing the the results of metatranscriptomics performed on CD, UC, and healthy ileum and colon*.* All box plots represent sample distribution with the median, min, max, first, and third quartiles. An interquartile range of 1.5 has been used to define outliers. Statistical differences between groups have been calculated by Analysis of variance (ANOVA) with Tukey's HSD (honestly significant difference) post-hoc test for multiple comparisons. Differences with adjusted p-value ≤ 0.05 were considered significant. For complete statistics, please refer to **Supplementary Table 2**.
